# Acute pancreatitis following semaglutide therapy: a case report

**DOI:** 10.1097/MS9.0000000000005389

**Published:** 2026-08-04

**Authors:** Rabia Khalid Alduraibi, Nagwa Abdurrahman Fattouta, Yosef Fahad Altowayan

**Affiliations:** Department of Endocrine and Diabetes, King Fahad Specialist Hospital, Buraydah, Saudi Arabia

**Keywords:** acute pancreatitis, drug-induced acute pancreatitis, GLP-1 RA, obesity, semaglutide

## Abstract

**Background:**

Acute pancreatitis (AP) is a potentially life-threatening condition that can result from various aetiologies. While gallstones and alcohol consumption are the most common causes, medications can also induce AP. In the absence of a clear cause, drug-induced acute pancreatitis (DIAP) may pose challenges for clinicians. This is especially true when addressing unusual and rare causes such as glucagon-like peptide-1 receptor agonists.

**Case Presentation:**

A 41-year-old Saudi man was admitted to the hospital with epigastric pain after receiving subcutaneous semaglutide for weight loss. Laboratory test results revealed AP.

**Conclusions:**

In conclusion, semaglutide-induced acute pancreatitis is a rare but serious adverse effect. Clinicians should be aware of this potential adverse effect, particularly in patients without a history of diabetes mellitus who are using the drug for weight loss. Early recognition and discontinuation of the drug are crucial for the management of this condition.

## Background

Acute pancreatitis (AP) is a potentially life-threatening condition that can result from various aetiologies. While gallstones (30–60%) and alcohol consumption (15–30%) are the most common causes, medications can also induce acute pancreatitis. Once the most common causes have been ruled out, it can be challenging to determine the cause of acute pancreatitis in a patient, as in our case.

Semaglutide, sold under the brand names Ozempic and Wegovy, is a popular glucagon-like peptide-1 receptor agonist (GLP-1 RA) that has been increasingly used in the management of type 2 diabetes mellitus and obesity following its FDA approval in June 2021[1]. Despite its effectiveness, there have been growing concerns regarding its association with AP. Most of the documented cases of AP have involved patients with diabetes or those with other known risk factors for pancreatitis^[^2–7^]^. In contrast, other recent studies have shown that, compared with nonusers, subcutaneous semaglutide use is not associated with an increased risk of AP^[^8–10^]^.

Here, we present a case of AP in a nondiabetic patient following the administration of subcutaneous semaglutide for weight loss. Subsequent to appropriate clinical evaluation, the patient was diagnosed with drug-induced acute pancreatitis (DIAP).

HIGHLIGHTS

- Semaglutide can rarely cause drug-induced acute pancreatitis (AP).
- Case report of AP in a 41-year-old male after taking semaglutide for weight loss.
- Early recognition and drug discontinuation are crucial for recovery.
- Clinicians should monitor nondiabetic patients using glucagon-like peptide-1 receptor agonist for obesity.

Therefore, with the increasing use of GLP-1 RAs, it is important for physicians to be aware of the potential risk of AP and to address the clinical implications of this adverse event through appropriate management strategies.

To the best of our knowledge, this is the first case report addressing the risk of AP associated with the use of GLP-1 RAs in nondiabetic patients.

## Case presentation

A 41-year-old male with a medical history of obesity (BMI 40) presented to the emergency department with a 24-hour episode of sudden-onset abdominal pain and nausea. The pain was described as severe and progressive, located in the right upper quadrant and radiating to the patient’s back. He did not present with any of the following symptoms: fever, chills, constipation, polyuria, polydipsia, or recent unintended weight loss. He did not report alcohol use, tobacco use, recreational drug use, or recent abdominal trauma. No similar episodes had previously been reported. There was no family history of dyslipidaemia, pancreatitis, or gallstones. The patient’s past surgical history was unremarkable. His only medication was semaglutide (1 mg), taken weekly for weight loss, which had been started 5 months earlier.

The initial assessment revealed a normal temperature of 36.6°C, a heart rate of 105 bpm, a blood pressure of 130/65 mmHg, a respiratory rate of 15 breaths per minute, and an oxygen saturation of 99% on room air. The physical examination revealed no xanthelasmas or skin eruptions. The abdomen was soft with diffuse tenderness and was not distended. The remainder of the examination was normal.

The initial laboratory tests revealed that his white blood cell count was 14.5 × 10^9^/L (reference range: 4–11.0 × 10^9^/L), hemoglobin level was 16.1 g/dL (reference range: 14–18 g/dL), sodium level was 134 mmol/L (reference range: 136–145 mmol/L), potassium level was 4.8 mmol/L (reference range: 3.5–5.3 mmol/L), chloride level was 103 mmol/L (reference range: 98–110 mmol/L), aspartate aminotransferase level was 25.1 U/L (reference range: 5–34 U/L), alanine aminotransferase level was 26.8 U/L (reference range: 5–56 U/L), triglyceride level was 0.9 mmol/L (reference range: 0.3–1.9 mmol/L), total bilirubin was 7.9 µmol/L (reference range: 0–8.6 µmol/L), and amylase level was 550 U/L (reference range: 25–125 U/L).

**Figure 1.**
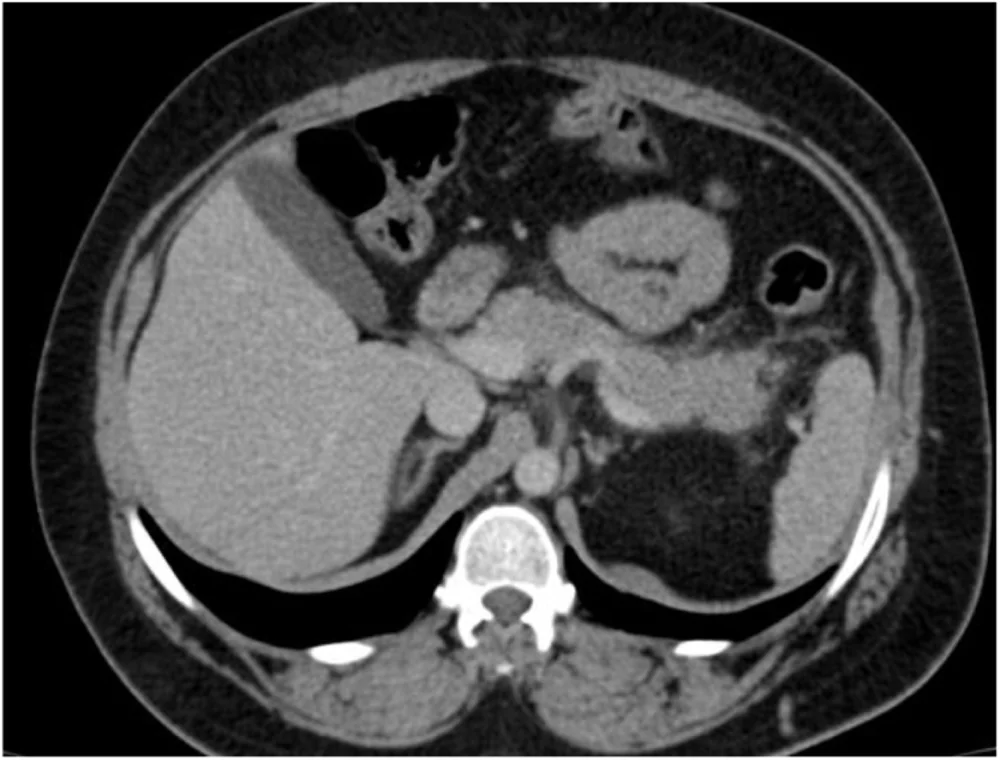
Computed tomography scan showing pancreatic edema.

Contrast-enhanced abdominal and pelvic computed tomography (CT) revealed a bulky, edematous distal body and tail of the pancreas, with associated peripancreatic fat stranding and fluid tracking extending to the perisplenic, left perirenal, and left paracolic regions. Multiple small regional lymph nodes measuring up to 14 mm were noted, and the findings are suggestive of acute pancreatitis, as shown in Figure 1. Furthermore, the patient underwent an abdominal ultrasound, the findings of which were normal.

The patient was diagnosed with acute pancreatitis (AP) on the basis of his characteristic abdominal pain and findings on computed tomography. To determine the cause of hisAP, an extensive history was taken and additional laboratory tests were performed, including IgG4, serum triglycerides, and serum calcium levels – all of which were normal.

After ruling out other potential causes and considering the timeline between his semaglutide use and the onset of pancreatitis, it became clear that semaglutide was the most likely cause. Treatment with intravenous fluids and pain medication was initiated immediately. All symptoms resolved the following day. He was also able to tolerate a solid diet without symptoms. The patient was informed of the diagnosis and advised to discontinue Ozempic. This is one of the few reported cases of Ozempic-induced acute pancreatitis. Therefore, it is important for physicians to be aware of the potential risk of semaglutide-induced acute pancreatitis.

## Discussion

DIAP is a potentially life-threatening medical condition that receives little attention due to its rarity. Although DIAP is generally considered rare, with an overall prevalence ranging from 0.1% to 2%, it is important to note that there have been documented cases of AP associated with various medications^[^11–13^]^. The most commonly implicated medications are analgesics and anti-inflammatory drugs, which account for approximately 30% of DIAP cases. Other common medications include antimicrobials, cardiovascular medications, and immunomodulatory drugs. There are no specific clinical diagnostic criteria for DIAP. However, a clinical confirmation of DIAP can be made when abdominal pain improves and pancreatic enzyme levels decline after discontinuation of the suspected drug.

The present case report describes a unique instance of GLP-1 RA-induced AP in a non-diabetic patient, which emphasizes the importance of considering this medication as a potential aetiological factor for AP in a broader patient population. GLP-1 RAs have been linked to an increased risk of AP and pancreatic adenocarcinoma in numerous studies. In contrast, AP was observed with semaglutide at rates comparable to placebo during the SUSTAIN-6 trial.

Semaglutide, a GLP-1 receptor agonist, is primarily used to manage type 2 diabetes mellitus, but it has also been prescribed for weight loss in nondiabetic patients because of its appetite-suppressing properties. The pathophysiology of GLP-1 receptor agonist-induced pancreatitis is not entirely clear. The drug may induce pancreatic duct constriction, leading to obstruction and subsequent inflammation. Another hypothesis is that GLP-1 receptor agonists stimulate pancreatic β-cell proliferation and increase pancreatic enzyme secretion, contributing to pancreatitis. The fact that our patient developed acute pancreatitis (AP) shortly after starting Ozempic, with no other apparent causes, supports a causal relationship. Although several case reports have documented an association between Ozempic and pancreatitis, especially in diabetic patients, this adverse effect has not been extensively studied in nondiabetic patients using the drug for weight loss. This represents a significant gap in the literature, given the increasing trend toward prescribing GLP-1 receptor agonists for weight management. Clinicians should be aware of this potential adverse effect and counsel their patients accordingly, especially those without a history of diabetes mellitus.

Early recognition of Ozempic-induced acute pancreatitis is crucial for timely intervention and the prevention of complications. In our case, discontinuation of Ozempic and supportive management led to a favorable outcome, highlighting the importance of prompt recognition and cessation of the drug.

## Conclusion

In conclusion, semaglutide-induced AP is a rare but serious adverse effect. Clinicians should be aware of this potential adverse effect, particularly in patients without a history of diabetes mellitus who are using the drug for weight loss. Early recognition and discontinuation of the drug are crucial for the management of this condition. Further research is needed to better understand the incidence of and risk factors for Ozempic-induced AP in nondiabetic populations.

## Data Availability

The data generated during this study are included in this published article.

## References

[1] Trujillo JM Nuffer W Smith BA. GLP-1 receptor agonists: an updated review of head-to-head clinical studies. Ther Adv Endocrinol Metab 2021;12:2042018821997320. 10.1177/2042018821997320 PMC7953228 33767808

[2] Alves C Batel-marques F Macedo AF. A meta-analysis of serious adverse events reported with exenatide and liraglutide: acute pancreatitis and cancer. Diabetes Res Clin Pract 2012;98:271–84. 10.1016/j.diabres.2012.09.008 23010561

[3] Elashoff M Matveyenko AV Gier B. Pancreatitis, pancreatic, and thyroid cancer with glucagon-like peptide-1-based therapies. Gastroenterology 2011;141:150–56. 10.1053/j.gastro.2011.02.018 PMC4404515 21334333

[4] Singh S Chang HY Richards TM. Glucagon-like peptide-1-based therapies and risk of hospitalization for acute pancreatitis in type 2 diabetes mellitus: a population-based matched case-control study. JAMA Intern Med 2013;173:534–39. 10.1001/jamainternmed.2013.2720 23440284

[5] Giorda CB Nada E Tartaglino B. A systematic review of acute pancreatitis as an adverse event of type 2 diabetes drugs: from hard facts to a balanced position. Diabetes Obes Metab 2014;16:1041–47. 10.1111/dom.12297 24702687

[6] Shetty R Basheer FT Poojari PG. Adverse drug reactions of GLP-1 agonists: a systematic review of case reports. Diabetes Metab Syndr 2022;16:102427. 10.1016/j.dsx.2022.102427 35217468

[7] Liu L Chen J Wang L. Association between different GLP-1 receptor agonists and gastrointestinal adverse reactions: a real-world disproportionality study based on FDA adverse event reporting system database. Front Endocrinol (Lausanne) 2022;13:1043789. 10.3389/fendo.2022.1043789 PMC9770009 36568085

[8] Giorda CB Sacerdote C Nada E. Incretin-based therapies and acute pancreatitis risk: a systematic review and meta-analysis of observational studies. Endocrine 2015;48:461–71. 10.1007/s12020-014-0386-8 25146552

[9] Storgaard H Cold F Gluud LL. Glucagon-like peptide-1 receptor agonists and risk of acute pancreatitis in patients with type 2 diabetes. Diabetes Obes Metab 2017;19:906–08. 10.1111/dom.12885 28105738

[10] Cao C Yang S Zhou Z. GLP-1 receptor agonists and pancreatic safety concerns in type 2 diabetic patients: data from cardiovascular outcome trials. Endocrine 2020;68:518–25. 10.1007/s12020-020-02223-6 32103407

[11] Jones MR Hall OM Kaye AM. Drug-induced acute pancreatitis: a review. Ochsner J 2015;15:45–51. PMC4365846 25829880

[12] Meczker Á Hanák L Párniczky A. Analysis of 1060 cases of drug-induced acute pancreatitis. Gastroenterology 2020;159:1958–61.e8. 10.1053/j.gastro.2020.07.016 32687926

[13] Nitsche C Maertin S Scheiber J. Drug-induced pancreatitis. Curr Gastroenterol Rep 2012;14:131–38. 10.1007/s11894-012-0245-9 22314811

